# Anti-Angiogenic miR-222, miR-195, and miR-21a Plasma Levels in T1DM Are Improved by Metformin Therapy, Thus Elucidating Its Cardioprotective Effect: The MERIT Study

**DOI:** 10.3390/ijms19103242

**Published:** 2018-10-19

**Authors:** Fahad W. Ahmed, Sherin Bakhashab, Inda T. Bastaman, Rachel E. Crossland, Michael Glanville, Jolanta U. Weaver

**Affiliations:** 1 Department of Diabetes, Queen Elizabeth Hospital, Gateshead, Newcastle Upon Tyne NE9 6SH, UK; nfahadahmed@gmail.com (F.W.A.); itashab@gmail.com (I.T.B.); 2 Institute of Cellular Medicine, Newcastle University, Newcastle Upon Tyne NE2 4HH, UK; sbakhashab@kau.edu.sa (S.B.); rachel.crossland@newcastle.ac.uk (R.E.C.); michael.glanville@newcastle.ac.uk (M.G.); 3 Department of Diabetes and Endocrinology, Royal Sussex County Hospital, Brighton BN2 5BE, UK; 4 Biochemistry Department, Faculty of Science, King Abdulaziz University, Jeddah P.O. Box 80218, Saudi Arabia; 5 Center of Innovation in Personalized Medicine, King Abdulaziz University, Jeddah P.O. Box 80216, Saudi Arabia; 6 Faculty of Medicine, Universitas Indonesia, Jakarta 10430, Indonesia; 7 Cardiovascular Research Centre, Newcastle University, Newcastle Upon Tyne NE2 4HH, UK

**Keywords:** T1DM, metformin, anti-angiogenic, miRs

## Abstract

Type 1 diabetes (T1DM) is associated with increased cardiovascular disease (CVD) and reduced life expectancy. We thus hypothesized that anti-angiogenic miRs are increased in T1DM, and the cardioprotective effect of metformin is mediated via reducing those miRs. In an open label, case-controlled study, 23 T1DM patients without CVD were treated with metformin for eight weeks (TG), matched with nine T1DM patients on standard treatment (SG) and 23 controls (CG). Plasma miR-222, miR-195, miR-21a and miR-126 were assayed by real-time RT-qPCR. The results were correlated with: endothelial function (RHI), circulating endothelial progenitor cells (cEPCs) (vascular repair marker, CD45^dim^CD34^+^VEGFR2^+^ cells) and circulating endothelial cells (cECs) (vascular injury marker, CD45^dim^CD34^+^CD133^-^CD144^+^ cells). miR-222, miR-195 and miR-21a were higher in T1DM than CG; *p* = 0.009, *p* < 0.0001, *p* = 0.0001, respectively. There was an inverse correlation between logmiR-222 and logRHI (*p* < 0.05) and a direct correlation between logmiR-222 and logCD34^+^ (*p* < 0.05) in TG. Metformin reduced miR-222, miR-195 and miR-21a levels in TG; *p* = 0.007, *p* = 0.002 *p* = 0.0012, respectively. miRs remained unchanged in SG. miR-126 was similar in all groups. There was a positive association between changes in logmiR-222 and logcECs after metformin in TG (*p* < 0.05). Anti-angiogenic miRs are increased in T1DM. Metformin has cardioprotective effects through downregulating miR-222, miR-195 and miR-21a, beyond improving glycemic control.

## 1. Introduction

Cardiovascular disease (CVD) remains the number one cause of death worldwide according to a 2016 World Health Organization report. The outcome of CVD management is affected by diabetes mellitus (DM), which results in a two- to four-fold increased risk of CVD [1]. The results of CVD interventions such as percutaneous coronary intervention (PCI) and coronary artery bypass graft in patients with DM are much worse than in non-diabetic individuals [2]. More recently, data have become available for type 1 DM (T1DM), not previously considered to be a high CVD risk, showing an increased risk for CVD, coronary heart disease (CHD), stroke and consequent all-cause mortality [3]. Overall, the largest percentage of the estimated loss in life expectancy was related to ischemic heart disease (36% in men, 31% in women) [3]. Thus, in order to improve the outcome of CVD patients and with T1DM in particular, it is paramount to understand the mechanisms involved in diabetes-associated CVD complications.

Metformin is the first hypoglycemic drug to display cardioprotective properties in type 2 diabetes as shown in a long-term randomized clinical trial [4]. A recent study in T1DM has shown that although progression of mean carotid intima media thickness (cIMT) was not significantly reduced with metformin, the maximal cIMT (a pre-specified tertiary outcome) was significantly reduced [5]. Furthermore, when metformin was used in patients with metabolic syndrome, the duration of post-PCI myocardial injury was decreased by seven days [6]. The pre-treatment of diabetic patients with metformin was found to be associated with reduced myocardial infarction (MI) size compared to non-metformin-treated patients [7]. Additionally, studies in diabetic animal models have suggested that metformin, in physiological doses, improves coronary blood flow following MI [8] and limits infarct size in type 2 diabetic rats [9]. It has also been shown that the vasculo-protective effects of metformin are independent of its anti-hyperglycemic and lipid lowering effects [10]. In animal models, metformin has prevented the development of pulmonary hypertension [10]. Metformin has also improved heart failure and survival in animals via activation of the AMPK pathway and its downstream mediators, endothelial nitric oxide synthase (eNOS) and peroxisome proliferator-activated receptor-gamma coactivator 1 (PGC-1). Furthermore, metformin-mediated cardioprotection was achieved independently of its effect on glucose levels [11].

Our recent work has shown that metformin, in vitro, improved angiogenesis by augmenting the expression of vascular endothelial growth factor A (VEGFA) and reducing the angiogenic inhibitors in CD34^+^ cells [12]. Furthermore, metformin upregulates VEGF receptors (VEGFR1/R2), fatty acid binding protein 4, ERK/mitogen-activated protein kinase signaling, chemokine ligand 8, lymphocyte antigen 96, Rho kinase 1, matrix metalloproteinase 16 (*MMP16*) and tissue factor inhibitor-2 in human umbilical endothelial cells exposed to hyperglycemia-hypoxia [13]. In our clinical trial in T1DM (MERIT Study), metformin improved levels of circulating endothelial progenitor cells (cEPCs), vascular repair markers (CD45^dim^CD34^+^VEGFR2^+^) and circulating endothelial cells (cECs), vascular injury markers (CD45^dim^CD34^+^CD144^+^CD133^-^), whilst being unchanged in diabetic control [14]. Other pleiotropic effects of metformin were documented in non-diabetic ApoE^−/−^ mice and showed amelioration of atherosclerosis and vascular senescence [15]. The cardioprotective mechanism behind metformin action is poorly understood in clinical practice. Therefore, if we understand its mode of action, this could facilitate the development of novel therapies for CVD in diabetes.

MicroRNAs (miRs) are a class of small, single-stranded, non-coding RNAs (~19–22 nucleotides) that function mainly in gene silencing by either repressing the translational process of target messenger RNAs (mRNAs) and/or enhancing target mRNAs’ degradation. Cell-derived miRs circulating in various body fluids are resistant to RNase activity and thus are suitable for assessing cell-to-cell communication and the monitoring of therapeutic interventions [16]. Numerous miRs have been identified as significant diagnostic or prognostic markers for CVD [17]. Multiple miRs, miR-1, miR-208a, miR-208b, miR-133a, miR-133b and miR-499, have been shown to be elevated in the plasma of patients with acute ischemic injury [18,19,20,21]. In addition to their diagnostic value, miRs have been identified to have prognostic value in patients with myocardial infarction, levels of miR-208b and miR-133a being correlated with increased mortality [22]. Elevated miR-1 levels are associated with a future risk of heart failure in patients with acute myocardial infarction [23]. miRs have been also identified as markers of the size of the myocardial injury [24]. However, our understanding of them is limited, and further work needs to be carried out to understand the mechanisms by which miRs play a role in CVD. We decided to explore the effect of metformin on four miRs, miR-222, miR-195, miR-21a and miR-126 based on their role as angiogenic and anti-angiogenic factors. These miRs appear to be deregulated in the plasma of diabetic individuals [25,26]. We therefore hypothesized that plasma levels of the anti-angiogenic miRs, miR-222, miR-195 and miR-21a are raised in T1DM, whilst the level of the angiogenic miR-126 is decreased, and the cardioprotective effect of metformin is mediated by modulating the levels of circulating miRs involved in angiogenesis; this is achieved beyond improving glycemic control.

## 2. Results

Patients’ characteristics have been discussed previously [14] and are provided in Table 1. Briefly, the treatment group (TG), standard group (SG) and healthy control group (CG) were well-matched for age, gender and blood pressure. Except for BMI, TG and SG were well-matched for duration of diabetes, HbA1c, baseline insulin dose, lipid profile and creatinine. In order to maintain unchanged diabetic control, insulin dose including insulin pump dose in the TG was significantly reduced (median 44 units, range 10.7–161, versus median 39 units, range 9.3–148, *p* = 0.0002). HbA1c remained unchanged between TG Visit 1 and TG Visit 2 (Table 1). Metabolic parameters (HbA1c, BMI, total cholesterol, triglyceride and blood pressure) were similar in both the TG and SG.

### 2.1. miRs

**Figure 1:**
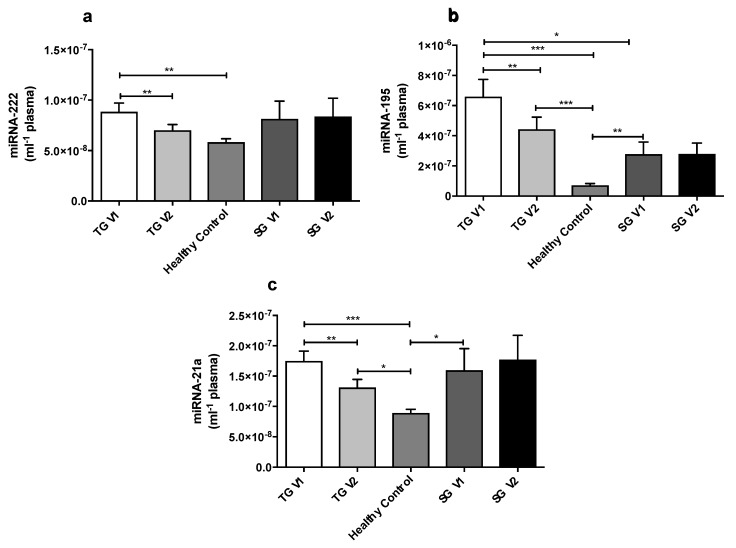
(**a**–**c**) A comparison of miR-222, miR-195 and miR-21a levels in plasma for all groups. At baseline (TG V1), miR-222, miR-195 and miR-21a levels were significantly upregulated in the TG compared to healthy controls (CG). The levels of miR-222, miR-195 and miR-21a were all significantly reduced in the TG after metformin therapy (TG V2). The results are presented as the mean ± SEM. Within the TG and SG, the comparison was analyzed using the paired Student *t*-test. An unpaired Student *t*-test was used to compare the healthy controls and patients’ groups. Treatment group pre-metformin (TG V1), treatment group post-metformin (TG V2), standard group pre-observation (SG V1) and standard group post-observation (SG V2). * *p* < 0.05, ** *p* < 0.01, *** *p* < 0.001.

At baseline, plasma miR-222, miR-195 and miR-21a levels were significantly higher in the TG V1 when compared with the CG *p* = 0.009, *p* < 0.0001, *p* = 0.0001 (Figure 1a–c), respectively. Plasma miR-195 and miR-21a levels were significantly higher in the SG V1 compared with the CG (*p* = 0.0012 and *p* = 0.02, respectively, Figure 1b,c), whilst the expression of miR-222 was not significantly different between the SG V1 and CG (Figure 1a). At baseline, plasma miR-222 and miR-21a levels were similar in TG V1 compared with SG V1 (pre-observation, Figure 1a,c). Whereas, plasma miR-195 level was significantly higher in TG V1 when compared with SG V1 (*p* = 0.03, Figure 1b), there was a significant difference in miR-195 and miR-21a plasma levels in SG V1 compared with CG (*p* = 0.0012 and *p* = 0.02, respectively, Figure 1b,c). There was no significant difference in miR-222 expression in the SG V1 and CG (Figure 1a).

### 2.2. The Correlation between miR-222 and Indices of Vascular Health

**Figure 2:**
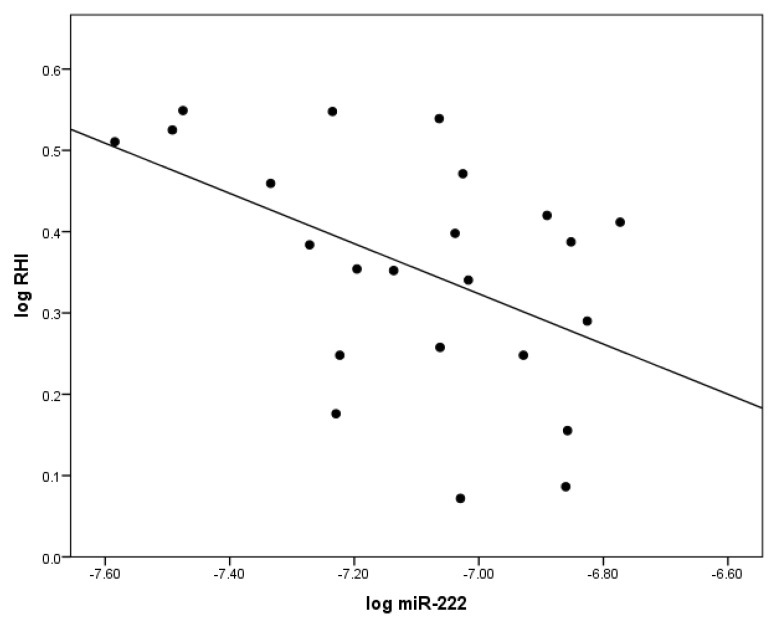
The significant correlation between levels of circulating miR-222 and indices of vascular health in patients with T1DM at baseline. LogmiR-222 was inversely correlated with endothelial function logRHI measured by the reactive hyperemia index (RHI). This relationship was demonstrated by Pearson correlation.

**Figure 3:**
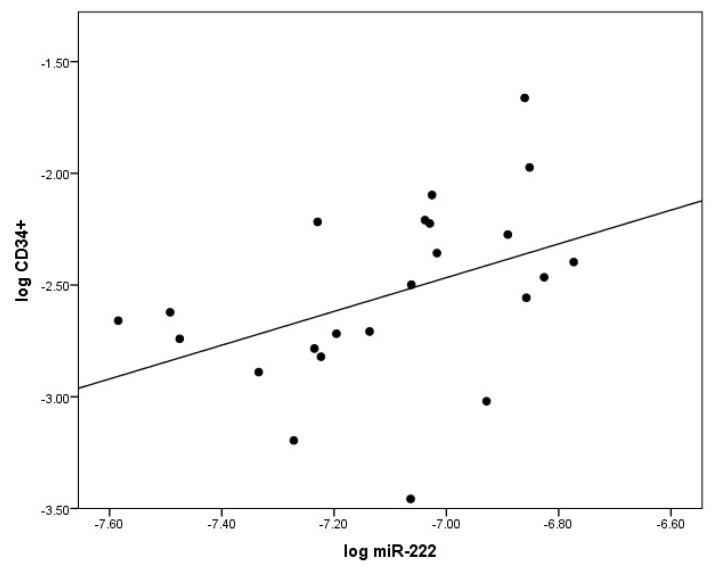
The significant correlation between levels of circulating miR-222 (logmiR222) and CD34^+^ cells (log CD34^+^) at baseline. CD34^+^ were analyzed by flow cytometry. The relationship was demonstrated by Pearson correlation.

At baseline, there was a significant inverse correlation between logmiR-222 and the flow-mediated dilatation reactive hyperemia index (RHI, clinical measure of endothelial function) log(RHI), *r* = −0.52; *p* < 0.05 in T1DM (Figure 2). Additionally, a direct positive correlation between logmiR-222 and logCD34^+^ (*r* = 0.42; *p* < 0.05) was observed in TG at baseline (Figure 3).

### 2.3. Anti-Angiogenic miRs Reduced by Metformin

#### 2.3.1. miR-222

**Figure 4:**
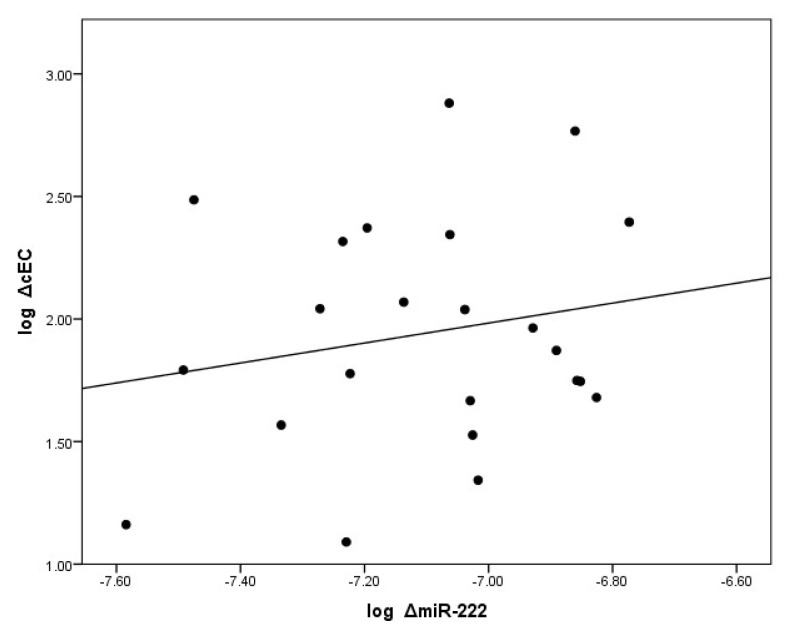
A direct correlation between the change (log delta) in levels of circulating miR-222 and the change (log delta) of circulating endothelial cells (cECs). cECs are defined by FACS as CD45^dim^, CD34^+^, CD 133^−^, CD144^+^ and delta cECs, an index of vascular damage. This relationship was demonstrated by Pearson correlation.

Eight weeks of metformin treatment significantly reduced the plasma levels of miR-222 in the TG, *p* = 0.007 (Figure 1a). After metformin treatment, plasma miR-222 levels were normalized in the TG in comparison to the GC. In the SG, plasma miR-222 remained unchanged after eight weeks of observation. There was also a direct positive correlation between changes in logmiR-222 and changes in logcEC, *r* = 0.42; *p* < 0.05, in the TG (Figure 4).

#### 2.3.2. miR-195

After eight weeks of metformin treatment, plasma miR-195 levels were significantly reduced in the TG, *p* = 0.002 (Figure 1b), whereas they remained unchanged after eight weeks of observation. No association was identified between changes in RHI, CD34^+^, cECs and miR-195 (*p* > 0.05).

#### 2.3.3. miR-21a

Eight weeks of metformin treatment significantly reduced plasma miR-21a levels in the TG *p* = 0.0012 (Figure 1c), whilst they remained unchanged after eight weeks of observation. No association was identified between changes in RHI, CD34^+^, cECs and miR-21a (*p* > 0.05).

#### 2.3.4. miR-126

**Figure 5:**
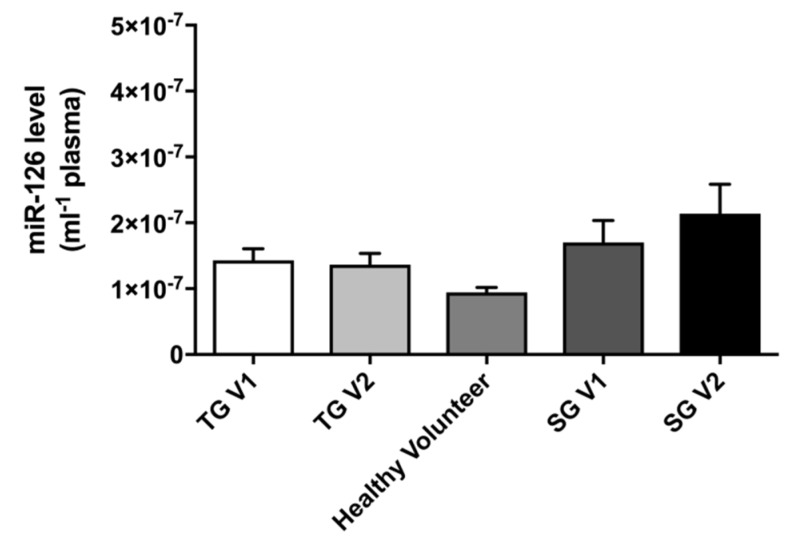
A comparison of the plasma levels of miR-126 (per mL) in all groups. The results are presented as the mean ± SEM. Treatment group pre-metformin (TG V1), treatment group post-metformin (TG V2), standard group pre-observation (SG V1) and standard group post-observation (SG V2).

Plasma miR-126 levels were similar in all the groups studied. In the TG, eight weeks of metformin treatment did not alter plasma miR-126 levels. Additionally, in the SG plasma, miR-126 remained unchanged after eight weeks of observation (Figure 5).

### 2.4. Anti-Angiogenic miRs Pathway Identification

**Figure 6:**
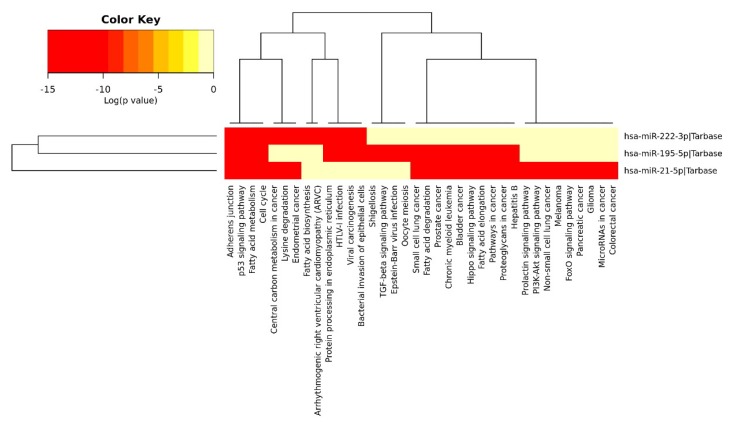
A heatmap showing the pathways altered by miR-222, miR-195 and miR-21a. The heatmap was generated with the DIANA miRPATH software, based on experimentally-validated miRNA interactions derived from the DIANA-TarBase. Axes on the attached dendrograms depict hierarchical clustering results for miRs and their pathways, respectively.

We predicted the biological pathways that would be affected by a significant decrease in the levels of the anti-angiogenic miRs miR-222 we studied, miR-195 and miR-21a (Figure 6), using the DIANA miRPATH (Version 3) software [27]. Analysis of the pathways demonstrated that the reduction in plasma levels of these miRs might affect angiogenesis, cellular proliferation, migration, adhesion and the cell cycle (Figure 6).

## 3. Discussion

We are the first group to establish that T1DM with good diabetic control and without cardiovascular disease is associated with increased anti-angiogenic miRs; miR-21a, miR-222, miR-195. Furthermore, we were able to validate that those miRs are associated with indices of vascular health or measures of health improvement, including the effect of metformin therapy. Below we discuss individual findings and what vascular effects of miRs studied by us are known in the literature.

### 3.1. miR-21a

#### 3.1.1. Clinical Studies

We found plasma miR-21a expression to be significantly higher in T1DM in both the TG V1 and SG V1 in comparison to the CG. Our data are concordant with another study, which demonstrated high plasma and urine miR-21a levels in T1DM versus controls [25]. Increased miR-21a levels in T1DM are considered to be an indicator of established or ongoing vascular damage [25]. An ongoing inflammatory process demonstrating a direct correlation of C-reactive protein (CRP) with urinary miR-21a is speculated to play a role in kidney damage [25]. Sala-Perez’s group has demonstrated that miR-21a levels were reduced in peripheral blood mononuclear cells (PBMC) collected from T1DM patients compared to healthy controls [28]. This might lead to a continued pro-inflammatory environment, maintained by apoptosis-resistant PBMCs [28].

It is of interest that plasma miR-21a levels in T2DM patients with a previous history of major cardiovascular events (MACE) were significantly higher compared to other T2DM patients [29]. Furthermore, in the same study, miR-21a levels in circulating pro-angiogenic cells (CACs) were the highest in T2DM patients with previous MACE compared to other T2DM patients. However, in another group of T2DM patients, the opposite effect was observed: low plasma miR-21a levels were recorded compared to controls [30]. Thus, the difference in miR-21a levels may be attributed to the presence or absence of MACE events, which occurred in the former group, but not in the latter [30].

In a study of subclinical atherosclerosis, plasma miR-21a levels were positively correlated with systolic, diastolic blood pressure, CRP and cIMT, but negatively correlated with nitric oxide (NO) and eNOS [31]. Similarly, in acute coronary syndrome, miR-21 levels were increased and correlated with CRP, age and visfatin, but negatively correlated with HDL-cholesterol [32].

#### 3.1.2. miR-21a and Fibrosis

In T1DM animal models, hyperglycemia-induced miR-21a expression in mesangial cells led to a reduction in phosphatase and tensin homolog (PTEN) expression, as well as a concomitant increase in AKT serine/threonine kinase (Akt) phosphorylation [33]. In the same study, miR-21a-enhanced high glucose-induced target of rapamycin complex 1 (TORC1) activity resulted in renal cell hypertrophy and increased fibronectin expression [33].

miR-21a, which is highly expressed in diabetic skin, may have a functional role in wound healing due to its effect on fibroblasts, which are involved in ulcer healing [34]. In contrast to the inhibitory effect of miR-21a on endothelial cell migration, it has been demonstrated to promote fibroblast migration, confirming a cell-specific effect for this miR [34]. Further evidence for a deleterious effect of miR-21a comes from a study in which it promoted the progression of fibrosis upon ischemia-reperfusion injury by regulating MMP-2 expression in the fibroblasts of the infarct zone by inhibition of PTEN, a direct target of miR-21a [35].

#### 3.1.3. miR-21a and Atherosclerosis

miR-21a has been shown to be involved in the pathogenesis of proliferative vascular disease such as atherosclerosis as it is upregulated in atherosclerotic plaques [36]. A study of additional pathway regulation confirmed miR-21a as a new angiogenesis inhibitor as it reduced cell migration and tubulogenesis through repression of RhoB [37]. Overexpression of miR-21a promotes inflammation by directly targeting and downregulating peroxisome proliferator-activated RT-qPCR receptor-α (PPAR-α), which increases the adhesion of monocytes to endothelial cells (ECs) and results in the increased pro-inflammatory responses of vascular endothelial cells [38].

In vivo and in vitro data have also suggested that hypoxia-induced miR-21a expression in EPCs leads to growth arrest and that an inhibitory effect of miR-21 on EPCs proliferation and angiogenesis is mediated by activating the transforming growth factor-beta (TGF-β) signaling pathway via downregulation of the WW domain-containing protein 1 (WWP1) [39]. In contrast, inhibition of miR-21a improved the migratory function of circulating angiogenic progenitor cells (APCs) [40], and its suppression improved EPCs’ survival by increasing high-mobility group A2 protein (HMGA2) [41]. The above-mentioned data thus confirm an inhibitory effect of miR-21a on angiogenesis and vascular repair.

#### 3.1.4. miR-21a and Vascular Smooth Muscle Cells

It is clear that miR-21a is tissue specific as its proliferative effect has been observed in VSMC, the developmental model of atherosclerosis. Upregulation of miR-21a in VSMCs has been shown to inhibit PTEN, thereby inhibiting apoptosis and promoting VSMC proliferation [42].

#### 3.1.5. The Effect of miR-21a on Other Cell Types

In the central nervous system, increased expression of miR-21a suppresses pro-apoptosis genes, Fas ligand (FasL), PTEN and programmed cell death protein 4 (PDCD4) in vitro, which are direct targets of it [43]; whilst in vivo treatment with antagomir-21 increases the expression of FasL and PTEN but has no effect on PDCD4. These results suggest that miR-21a plays an important role in limiting secondary cell death following spinal cord injury and that its protective effects might be related to its regulation of pro-apoptotic genes [43]. Therefore, miR-21a expression in different cells with their own unique function/genetic component is likely to play a role in the development of vascular disease. We believe that high plasma miR-21a levels in our T1DM cohort confirm an ongoing process of diabetes-related vascular pathogenesis and its clinical complications.

In our MERIT Study, we have shown for the first time that metformin decreases miR-21a plasma levels in T1DM patients independent of improving glycemic control, whilst a standard treatment group (SG) without metformin over eight weeks’ follow-up did not show any change in plasma miR-21a levels. In a high-fat dietary rat model, metformin ameliorates skeletal muscle insulin resistance by inhibiting miR-21a expression in a dose-dependent manner [44]. In this model, miR-21a expression correlates directly with homeostatic model assessment-insulin resistance (HOMA-IR, index of insulin resistance) and inversely with insulin sensitivity (HOMA-ISI) [44].

The indirect evidence of metformin’s effect on miR-21a downregulation has come from suppression of the TGF-β pathway in cardiac fibroblasts [45]. In ECs, metformin has been demonstrated to inhibit the nuclear factor-κB signaling pathway and activate AMPK-induced PTEN expression, leading to decreased inflammatory response in VSMCs [46].

The flow diagram in Supplementary Figure S1 provides a summary of miR-21a action in the development of vascular disease in T1DM (Supplementary Figure S1a) and the protective effect of metformin (Supplementary Figure S1b) based on the evidence discussed above.

### 3.2. miR-222

#### Clinical Studies

miR-222 and miR-221 are located on the same gene, separated by a distance of 726 base pairs. They both play a vital role in vascular homeostasis. Most studies have evaluated mir-221 and miR-222 concurrently. However, in our study, the miR-221 *Cq* (quantification cycle) value was high (>35.00). This indicates that plasma levels of this miRNA are very low and cannot be reliably measured in plasma. It also has a high coefficient of variation (CV%). Therefore, we have only evaluated miR-222. We have demonstrated that plasma levels of miR-222 are significantly higher in T1DM individuals (TG V1) compared to the CG and are similar to those in SG V1. However, although miR-222 levels were higher in SG V1 compared to CG, this was not statistically significant, probably due to a smaller number of subjects in the SG. Overall, our miR-222 results are concordant with other research, which has demonstrated that miR-222 levels are elevated in T2DM [26]. Individuals with hypertension, metabolic syndrome, insulin resistance and CVD may also have raised plasma miR-222 levels [47].

Our work is the first study to show that miR-222 is negatively correlated with in vivo endothelial function (RHI), thus validating its anti-angiogenic properties in clinical practice consistent with its negative effect on the endothelium.

As the miR-222 cluster is highly expressed in quiescent ECs, this suggests that miRNA has a critical role in regulating the development and function of the vascular endothelium. However, it is important to remember that the role of miR-221/222 in vascular ECs greatly varies depending on the subject’s developmental stage and microenvironment.

In an inflammatory microenvironment, miR-222 has been demonstrated to be the main regulator of vascular homeostasis by negatively regulating the signal transducer and activator of transcription 5A (STAT5A) [48]. STAT5A is a transcription factor that regulates the expression of genes involved in cell proliferation, survival and differentiation [49]. The other mechanism of action of miR-222 is via reduction of eNOS protein in ECs [50]. Both miR-222 and miR-221 are highly expressed in human aortic ECs undergoing senescence. miR-222 negatively affects eNOS activity and synthesis in these cells [51].

Other anti-angiogenic mechanisms demonstrated by the miR-222 cluster were reported by Poliseno et al. They observed that miR-222 attenuated the angiogenic function of the stem cell factor by affecting the expression of c-kit [52]. This anti-angiogenic effect appears to be cell specific, as its overexpression in ECs reduces angiogenesis, whilst in cancer, cells increased angiogenesis [53]. Further evidence for a cell-specific action of miR-222 is derived from experiments using VSMCs. miR-222 stimulated proliferation, migration and attenuated apoptosis of VSMCs, thus displaying pro-atherogenic properties [54]. The opposing cellular effect of miR-221/222 on proliferation, migration and apoptosis of ECs and VSMCs allows miR-221/222 to promote neointimal formation while inhibiting re-endothelialization after vascular injury [54]. Vascular expression of miR-221/222 was upregulated in initial atherogenic stages, causing inhibition of angiogenic recruitment of ECs and increasing endothelial dysfunction and EC apoptosis. These miRNAs stimulated VSMCs and switched from the VSMC “contractile” phenotype to the “synthetic” phenotype associated with the induction of proliferation and motility [54].

Further tissue-specific effects of miR-222 are related to the expression profiles of its target genes [54]. At the site of vascular injury, platelets and ECs release platelet-derived growth factor (PDGF), which induces expression of miR-222 and miR-221 [55]. Increased expression of miR-222/miR-221 reduces levels of p27^kip1^ [54], which is known to attenuate atherosclerosis development, whilst reduced expression of p27^kip1^ in aortic tissue led to the development of atherosclerotic plaque [56].

Another important finding in our study was the positive correlation between miR-222 levels and CD34^+^ progenitor cells, but not with differentiated cEPCs (CD45^dim^ CD34^+^ VEGFR2^+^). As there was no correlation between miR-222 and CD34^+^ in CG, we can infer that a diabetic environment contributed to the association between plasma miR-222 levels and undifferentiated CD34^+^ cells. Our observation is supported by research from others where miR-222 has been found to be highly expressed in CD34^+^ hematopoietic cells from both umbilical cord blood and bone marrow [53]. Furthermore, miR-222 reduced the differentiation of hematopoietic progenitor cells [57]. It has been suggested that this mechanism is mediated through the c-kit and/or eNOS pathways [50,58].

All the above evidence indicates miR-222 to be a key player in vascular biology through its contribution to vascular remodeling, an adaptive process involving phenotypic and behavioral changes in vascular cells in response to vascular injury. One can conclude that the miR-222 cluster is primarily responsible for maintaining endothelial integrity and supporting the quiescent EC phenotype. Vascular expression of miR-221/222 is upregulated in the initial atherogenic stages causing the inhibition of angiogenic recruitment of ECs and increasing EC dysfunction and apoptosis, whilst in proliferative vascular diseases such as atherosclerosis, pathological vascular remodeling plays a prominent role.

In atherosclerotic vessels, miR-221/222 drive neointimal formation. Both miRNAs contribute to the atherogenic calcification of VSMCs. In advanced plaques, chronic inflammation downregulates miR-221/222 expression in ECs, which in turn may activate intralesional neo-angiogenesis [47]. Thus, it appears that miR-222 is involved in both physiological and atherosclerotic vascular remodeling.

To our knowledge, we are the first group to show that eight weeks of metformin treatment reduces plasma miR-222 levels in patients with T1DM. Furthermore, miR-222 levels were reduced to levels similar to those in healthy controls.

Our findings are concordant with two previously published studies in T2DM, which showed that metformin treatment, whilst lowering blood glucose concentration, reduces plasma miR-222 levels and miR-222 expression in the internal mammary artery [26,59]. In the former study, miR-222 reduction coincided not only with improved diabetic control, but also correlated with improved insulin sensitivity during the insulin clamp. In keeping with this relationship, the same group has shown that miR-222 levels increased following infusion of an intralipid-heparin mixture suggesting that miR-222 is related to insulin resistance [26]. Therefore, decreased miR-222 expression in the diabetic microenvironment may enhance the angiogenic pathway and vascular repair. In our study, we aimed to keep diabetic control unchanged, in order to remove the possibility of metabolic improvement as the mechanism behind metformin action on miR reduction. Thus, we can conclude that metformin has cardioprotective effects beyond its effect on glucose levels. Another example of cardioprotection by reducing miR-222 levels comes from using cholesterol-lowering agents. In patients with coronary artery disease, treatment with the drug, atorvastatin (a well-documented cardioprotective, 3-hydroxy-3-methylglutaryl coenzyme A (HMG CoA) reductase inhibitor) reduces miR-222 expression in EPCs, leading to their increased mobilization via the eNOS-dependent pathway, confirming the mechanism suggested by our study [58].

To further confirm our conclusion, we have shown an additional positive correlation between the reduction in plasma miR-222 levels and a reduction in circulating EC levels, a marker of endothelial injury, supporting evidence for metformin’s cardioprotective effects on miR-222 and endothelial homeostasis beyond improving diabetic control.

Thus, we can infer that metformin decreases vascular damage by improving miR-222 levels. Further work using miR-222 knock-in and knock-down in a culture microenvironment should be conducted to evaluate downstream pathways. The flow diagram in Supplementary Figure S2 summarizes miR-222 action in the development of vascular disease in T1DM (Supplementary Figure S2a) and the protective effect of metformin (Supplementary Figure S2b) based on the evidence discussed above.

### 3.3. miR-195

To our knowledge, we are the first group to show that plasma levels of miR-195 are significantly higher in T1DM patients in the TG V1 and SG V1 compared to the CG. When the TG V1 and SG V1 groups were compared, miR-195 levels were significantly higher in the TG probably due to the smaller number of subjects in the SG.

As miR-195 has been shown to be elevated in patients with acute myocardial ischemia, it has been defined as a biomarker of CVD [60]. In contrast to our data, miR-195 levels have also been observed to be significantly lower in T2DM [26]. The difference in the results might be multifactorial, including studying T2DM rather than T1DM [26].

In the animal model of T1DM, miR-195 has been linked to diabetes-related complications [61]. It has been demonstrated that miR-195 plays a major role in diabetic retinopathy by downregulating sirtuin 1 (SIRT1) and anti-apoptotic protein [61]. In the animal model of both T1DM (streptozotocin) and T2DM (db/db mouse), increased miR-195 reduced SIRT-1 levels in mouse hearts, whilst knocking down miR-195 expression, decreasing oxidative stress, increasing myocardial capillary density and improving maximal coronary blood flow [62].

In experimental gerontology, abrogation of age-induced miR-195-rejuvenated senescent mesenchymal stem cells (MSCs) has been achieved by reactivating telomerase [63]. This was achieved by silencing miR-195 in old MSCs by transfection of a miR-195 inhibitor and significantly restoring anti-aging factor expression including telomerase reverse transcriptase (Tert) and Sirt1, as well as phosphorylation of Akt and forkhead box protein O1 (FOXO1). Furthermore, transplantation of old MSCs with knocked-out miR-195 reduced myocardial infarct size and improved left ventricular function, due to increased MSC proliferative capabilities [63].

#### miR-195 and Metabolism

The additional metabolic effects of increased miR-195 levels have been studied in hepatocytes [64]. Overexpression of miR-195 by saturated fatty acids or a high fat diet impaired the insulin signaling cascade and glycogen synthesis in HepG2 cells. Furthermore, miR-195 directly suppresses the expression of insulin receptor (INSR), by targeting the INSR-3’UTR [64]. Thus, based on the evidence mentioned above, we can infer that high miR-195 levels in T1DM can be linked to the development of diabetes-related complications and CVD.

To our knowledge, we are the first group to demonstrate that eight weeks of metformin therapy significantly reduces miR-195 levels in T1DM patients. In tissue cultures, metformin has been shown to attenuate hyperglycemia-related endothelial senescence due to partly restoring SIRT-1 expression [65]. Moreover, restoring SIRT1 expression has been demonstrated to play a protective role in diabetes vasculopathy [66]. Thus, based on the above-discussed evidence, we hypothesize that metformin’s effect in reducing miR-195 expression may lead to a decreased risk of developing diabetes-related complications, by improving SIRT-1 expression. In our study, diabetic control was kept purposefully unchanged, so metformin displayed properties beyond improving glycemia. As it is well established that hyperglycemia itself decreases SIRT1 expression, metformin may act in two ways, via miR-195 reduction and by improving glycemic control (thus also indirectly improving SIRT-1). Therefore, it may be an adjunct cardioprotective therapy with dual action. Supplementary Figure S3a describes the schematic relationship between miR-195 and factors involved in angiogenesis and Figure S3b the effect of metformin on the interactions described in the evidence discussed above.

### 3.4. miR-126

Our results showing that metformin treatment did not change plasma miR-126 levels are not surprising. This is in line with data from a T2DM study in which three months of metformin treatment did not alter the expression of miR-126 plasma levels [26]. Therefore, we can conclude that metformin is unlikely to affect plasma miR-126 levels.

### 3.5. MicroRNA Pathway Identification (DIANA Tools miRPath)

We explored which pathways may be affected by the three significantly reduced (miR-21a, miR-222 and miR-195) anti-angiogenic miRs using DIANA Tools miRPath (Figure 5) [27]. Pathway analysis demonstrated that in T1DM angiogenesis, cell proliferation, migration, adhesion, cell cycle and apoptosis pathways are all likely to be dysregulated in T1DM. To support our findings that metformin directly or indirectly affects miR levels, it has been established that metformin treatment increases levels of the microRNA-processing protein DICER1 in patients with DM by upregulating it through a post-transcriptional mechanism involving the RNA-binding protein AUF1. Metformin treatment decreases cellular senescence in several senescence models in a DICER1-dependent manner [67]. Additionally, our previous study on HUVEC induced by hyperglycemia-hypoxia proved that metformin significantly enhances cell migration and inhibits apoptosis [13].

Thus, our data have yielded important information regarding angiogenic signals generated by metformin cardioprotective therapy. Our work can be meaningfully extended by exploring miR targets and downstream protein expression for the design of future therapeutic agents. The gain and loss of miR function in vitro and in vivo experiments will yield information regarding individual miRs and their functions. This will be important in not only exploring the function of circulatory biomarkers, but also in identifying prognostic markers and future treatment targets.

In summary, well-controlled T1DM is associated with increased circulating levels of the anti-angiogenic miRs; miR-21a, miR-222 and miR-195, confirming an increased cardiovascular risk in this disease group. miR-222 expression correlates with endothelial dysfunction, vascular injury and CD34^+^ cell levels. The cardioprotective effect of metformin may be mediated by downregulation of these three anti-angiogenic miRs.

## 4. Materials and Methods

### 4.1. MERIT Study

Plasma samples were used for miRs analysis from our patients and healthy controls who participated in the MERIT Study. Blood samples were collected at baseline (i.e., pre-treatment or pre-observation period) and at the end of the study.

The clinical study has been discussed previously [14]. In summary, 23 patients, aged 40.6 ± 4 years old with T1DM (HbA1c 7.3% or 56.4 mmol/mol) were recruited, as TG was treated with metformin. We included 9, age (47 ± 14 years), sex matched T1DM patients as SG (Table 1). The study protocol consisted of two phases: a run-in phase of 6 weeks ensured stable glucose control, which was followed by a treatment/observation phase. Metformin was given for 8 weeks to TG with a dose titrated up to a maximum of 1 g twice a day over 2–3 weeks or to the highest tolerated dose. The SG underwent similar follow-up except for metformin treatment. The dose of insulin was adjusted during the study to maintain unchanged the control confirmed by HbA1c and continuous glucose monitoring [14]. Furthermore, the TG was compared with 23 age 39.7 ± 10.3 year and gender-matched non-diabetic HC.

The clinical trial was approved by the NHS Health Research Authority, NRES Committee North East-Sunderland, UK (Research Ethics Committee Reference Number 12/NE/0044) on 29th of March 2012 and all subjects had given informed written consent. The study was performed in compliance with the Helsinki Declaration.

### 4.2. Vascular Function Measurements by Peripheral Tonometry (Endo-PAT^TM^)

An EndoPAT^TM^ 2000 (Itamar Medical Ltd., Keisarya, Israel) device was used to assess endothelial function (flow-mediated dilatation). This was measured before metformin therapy and at the end of the study. EndoPAT measurements were taken in the active intervention group only in the morning after overnight fasting as per Itamar recommendation. The data were analyzed by the EndoPAT software and presented as RHI, that is post-occlusion to pre-occlusion ratio. An RHI value of 1.67 or above was considered as normal [68].

### 4.3. Endothelial Progenitor Cells 

Peripheral blood samples were collected in EDTA tubes before and after the intervention phase for the TG and SG and at baseline for the CG. The first 4 mL of collected blood were not used for cEPC analysis to avoid spurious results. Blood samples were processed within 4 h of collection.

### 4.4. Flow Cytometric Evaluation of Circulatory Endothelial Progenitor Cells and of Circulatory Endothelial Cells

Whole blood (100 µL) was incubated with 5 µL of V500 CD45 (BD Bioscience, San Jose, CA, USA), 20 µL of PerCP-Cy5.5 CD34 (BD Bioscience, San Jose, CA, USA), 5 µL of PE VEGFR-2 (R & D Systems, Minneapolis, MN, USA ), 5 µL APC CD133 (Miltenyi Biotic Inc., Bergisch Gladbach, Germany) and 10 µL of FITC CD144 (BD Bioscience, San Jose, CA, USA) for 30 min. Subsequently, 2 mL of PharmLyse (BD Bioscience, San Jose, CA, USA) were used to lyse the red cells. The sample was then analyzed by flow cytometry on a BD FACS Canto™ II system and the results data processed using BD FACSDiva™ software as described previously [14].

### 4.5. Plasma Collection for miR Studies

Platelet-free plasma (PFP) was extracted from peripheral blood, collected in EDTA vacutainer tubes from subjects after an overnight fast. Samples were centrifuged for 15 min at 500× *g*. The upper, clear fraction (platelet-rich plasma, PRP) was collected without disturbing the bottom cellular layer and transferred to 1.5-mL polypropylene tubes. These PRP samples were further centrifuged for 5 min at 13,000× *g* in a microcentrifuge. After centrifugation, the clarified PFP was collected and stored at −80 °C for subsequent analysis.

### 4.6. miR Level Assay Using Reverse Transcription Quantitative Real-Time Polymerase Chain Reaction

miR levels were assayed directly from PFP using the TaqMan^TM^ RT-qPCR platform (Life Technologies, Paisley, UK) following the Minimum Information for Publication of Quantitative Real-Time PCR Experiments (MIQE) guidelines [69]. Control samples (RT control) for each miR were prepared in parallel for each patient, and the reverse transcriptase enzyme was omitted from the reaction mix to avoid false-positive results due to DNA contamination. The PFP were diluted 1:3 with nuclease-free water (Ambion, Paisley, UK). The diluted PFP were heated to 95 °C for 10 min then cooled to 4 °C. The TaqMan^TM^ MicroRNA Reverse Transcription Kit (Life Technologies, Paisley, UK) was used to prepare the reverse transcription reactions according to the manufacturer’s instructions.

Two-microliter aliquots of the reverse transcription reaction product were combined with 18 µL of PCR master mix (10 µL 2× TaqMan Fast Advanced master mix (Life Technologies, Paisley, UK), 1 µL 20× miRNA assay (Life Technologies, Paisley, UK), 7 µL nuclease-free water). The amplification reactions were performed using the Applied Biosystems 7900HT Fast Real-Time PCR system (Life Technologies, Paisley, UK) as follows: 50 °C for 2 min, 95 °C for 20 s followed by 50 cycles of 95 °C for 1 s and 60 °C for 20 s. The samples were tested in triplicate.

The Cy0 method was used to quantify the expression of miRNA using the PCR kinetics equation described by others, *R*_0_ *= R_Cq_ x 2^−Cq^* [70], where *R*_0_ and *R_Cq_* are initial fluorescence and fluorescence at *Cq* (quantification cycle) values, respectively. The advantage of the Cy0 method is to minimize the variation introduced due to slight inhibition. This inhibition may be caused by the carry-over of reagents during the RNA extraction. Such inhibition leads to the shifting of the amplification curve to the right, generating higher *Cq* values than those found under optimal amplification conditions and thus underestimating the target amount.

### 4.7. Statistical Analysis

The results are presented as the mean ± SEM unless stated otherwise. Log transformation of miR expression was carried out before performing the comparisons and correlations. The unpaired Student *t*-test or the Mann–Whitney test was used for the comparison between the groups. The paired Student *t*-test or Wilcoxon signed rank test was used within the intervention/observation group depending on the type of data distribution. Statistical significance was accepted at *p* < 0.05 (two-tailed significance). All analyses were conducted using IBM^TM^ SPSS^TM^ software Version 23 (SPSS^TM^ Inc., Armonk, NY, USA).

## 5. Conclusions

Metformin, with its proven life-extending interventions, can be considered as a pro-angiogenic and rejuvenating agent by affecting the differential patterns of miR expression.

## Figures and Tables

**Table 1:** Subjects’ clinical and metabolic characteristics.

Characteristics	TG (*n* = 23)		*p*-Value TG V1 vs. V2	HC (*n* = 23)	*p*-Value HC vs. TG V1	SG (*n* = 9)		*p*-Value SG V1 vs. V2	*p*-Value SG V1 vs. TG V1
	TG V1	TG V2				SG V1	SG V2		
**Age year**	46 ± 13	−	−	46 ± 12.6	1	47.4 ± 13.6	−	−	0.8
**Sex M/F n**	11/12	−	−	11/12	−	5/4	−	−	−
**DOD years**	23 ± 13.6	−	−	−	−	23.7 ± 14.1	−	−	0.9
**BMI kg/m^2^**	28.7 (24–32)	29 (23–32)	>0.05	26.2 ± 4.7	0.1	23.8 (22–27)	23.7 (21.3–27.1)	0.3	<0.05
**Systolic BP mmHg**	125 ± 10.8	121 ± 14	0.2	119.4 ± 9	0.2	132.8 ± 6.2	130.8 ± 12.1	0.7	0.05
**Diastolic BP mmHg**	76.2 ± 9.2	74 ± 7	0.1	75.7 ± 9	0.9	77 ± 8.2	72.9 ± 3.6	0.4	0.8
**HbA1c mmol/mol**	56.9 ± 10.5	55.9 ± 8.5	0.5	34.8 ± 2.9	<0.0001	58.6 ± 7.4	59 ± 9	0.7	0.6
**HbA1c %**	7.3 ± 0.9	7.3 ± 0.8	0.6	5.3 ± 0.3	<0.0001	7.5 ± 0.70	7.5 ± 0.8	0.6	0.6
**Insulin dose units**	44 (20–69)	39 (18–66)	<0.001	−	−	52.3 ± 11	52.9 ± 11	0.5	0.4
**Smoking Y/E/N**	4/2/17	−	−	0/0/23		2/1/6	−	−	−
**Total cholesterol mmol/L**	4.5 ± 0.8	4.4 ± 1	0.2	4.96 ± 0.8	0.1	4.8 ± 1.3	4.9 ± 1.4	0.8	0.7
**Triglyceride mmol/L**	0.9 ± 0.4	0.9 ± 0.4	0.9	1.5 ± 0.9	0.008	0.7 ± 0.32	0.7 ± 0.3	0.6	0.2
**HDL-cholesterol mmol/L**	1.8 ± 0.5	1.6 ± 0.4	<0.05	1.6 ± 0.4	0.1	1.9 ± 0.6	2.1 ± 0.6	0.4	0.5
**Creatinine µmol/L**	73 (68–94)	70 (63–77)	0.01	78 (70–87)	0.3	75 (65–87)	77 (62.8–83.5)	0.7	0.7
**WCC cells/mL**	6.4 ± 2.4	6.3 ± 2	0.7	6.3 ± 1.6	0.9	5.8 ± 1.5	5.6 ± 1.7	0.9	0.5

Values are given as mean ± SD or * median (interquartile range (IQ). kg, kilogram; BMI, body mass index; BP, blood pressure; M, male; F, female; DOD, duration of diabetes; Y, yes; E, ex-smoker; N, no. TG V1, pre-treatment; TG V2, post-treatment; SG V1, pre-observation; SG V2, post-observation; V1, visit 1; V2, visit 2; WCC, white cell count. * HbA1c TG V2 vs. SG V2 *p* = 0.6.

## Supplementary Materials

Supplementary materials can be found at http://www.mdpi.com/1422-0067/19/10/3242/s1.

## Abbreviations

Akt	AKT serine/threonine kinase
APC	Angiogenic progenitor cell
BMI	Body mass index
CAC	Circulating pro-angiogenic cell
cEC	Circulating endothelial cell
cEPC	Circulating endothelial progenitor cell
CG	Healthy control group
CHD	Coronary heart disease
cIMT	Carotid intima media thickness
CPR	C reactive protein
Cq	Quantification cycle
CVD	Cardiovascular disease
EC	Endothelial cell
eNOS	Endothelial NO synthase
FasL	Fas ligand
FOXO1	Forkhead box protein O1
HMGA2	High-mobility group A2
HOMA	Homeostatic model assessment
INSR	Insulin receptor
MACE	Major cardiovascular events
MI	Myocardial infarction
miR	MicroRNA
MMP	Matrix metalloproteinase
mRNA	Messenger RNA
MSC	Mesenchymal stem cell
NO	Nitric oxide
PBMC	Peripheral blood mononuclear cells
PCI	Percutaneous coronary intervention
PDCD4	Programmed cell death protein 4
PDGF	Platelet derived growth factor
PFP	Platelet free plasma
PPAR-α	Peroxisome proliferators activated receptor-α
PRP	Platelet rich plasma
PTEN	Phosphatase and tensin homolog
RHI	Reactive hyperemia index
RT-qPCR	Reverse transcription quantitative real-time polymerase chain reaction
SG	Standard group
SIRT1	Sirtuin 1
STAT5A	Signal transducer and activator of transcription 5A
T1DM	Type 1 diabetes
Tert	Telomerase reverse transcriptase
TG	Treatment group
TGF-β	Transforming growth factor-beta
TORC1	Target of rapamycin complex 1
VEGF	Vascular endothelial growth factor
VSMC	Vascular smooth muscle cell
WWP1	WW domain-containing protein 1

## References

[1] Nolan C.J., Damm P., Prentki M. (2011). Type 2 diabetes across generations: From pathophysiology to prevention and management. Lancet.

[2] Piccolo R., Galasso G., Iversen A.Z., Eitel I., Dominguez-Rodriguez A., Gu Y.L., de Smet B.J., Mahmoud K.D., Abreu-Gonzalez P., Trimarco B. (2014). Effects of baseline coronary occlusion and diabetes mellitus in patients with ST-segment elevation myocardial infarction undergoing primary percutaneous coronary intervention. Am. J. Cardiol..

[3] Livingstone S.J., Levin D., Looker H.C., Lindsay R.S., Wild S.H., Joss N., Leese G., Leslie P., McCrimmon R.J., Metcalfe W. (2015). Estimated life expectancy in a Scottish cohort with type 1 diabetes, 2008–2010. JAMA.

[4] UK Prospective Diabetes Study (UKPDS) Group (1998). Effect of intensive blood-glucose control with metformin on complications in overweight patients with type 2 diabetes (UKPDS 34). Lancet.

[5] Petrie J.R., Chaturvedi N., Ford I., Brouwers M., Greenlaw N., Tillin T., Hramiak I., Hughes A.D., Jenkins A.J., Klein B.E.K. (2017). Cardiovascular and metabolic effects of metformin in patients with type 1 diabetes (REMOVAL): A double-blind, randomised, placebo-controlled trial. Lancet Diabetes Endocrinol..

[6] Li J., Xu J.P., Zhao X.Z., Sun X.J., Xu Z.W., Song S.J. (2014). Protective effect of metformin on myocardial injury in metabolic syndrome patients following percutaneous coronary intervention. Cardiology.

[7] Lexis C.P., Wieringa W.G., Hiemstra B., van Deursen V.M., Lipsic E., van der Harst P., van Veldhuisen D.J., van der Horst I.C. (2014). Chronic metformin treatment is associated with reduced myocardial infarct size in diabetic patients with ST-segment elevation myocardial infarction. Cardiovasc. Drugs Ther..

[8] Legtenberg R.J., Houston R.J., Oeseburg B., Smits P. (2002). Metformin improves cardiac functional recovery after ischemia in rats. Horm. Metab. Res..

[9] Bhamra G.S., Hausenloy D.J., Davidson S.M., Carr R.D., Paiva M., Wynne A.M., Mocanu M.M., Yellon D.M. (2008). Metformin protects the ischemic heart by the Akt-mediated inhibition of mitochondrial permeability transition pore opening. Basic Res. Cardiol..

[10] Agard C., Rolli-Derkinderen M., Dumas-de-La-Roque E., Rio M., Sagan C., Savineau J.P., Loirand G., Pacaud P. (2009). Protective role of the antidiabetic drug metformin against chronic experimental pulmonary hypertension. Br. J. Pharmacol..

[11] Calvert J.W., Gundewar S., Jha S., Greer J.J., Bestermann W.H., Tian R., Lefer D.J. (2008). Acute metformin therapy confers cardioprotection against myocardial infarction via AMPK-eNOS-mediated signaling. Diabetes.

[12] Bakhashab S., Ahmed F.W., Schulten H.J., Bashir A., Karim S., Al-Malki A.L., Gari M.A., Abuzenadah A.M., Chaudhary A.G., Alqahtani M.H. (2016). Metformin improves the angiogenic potential of human CD34(+) cells co-incident with downregulating CXCL10 and TIMP1 gene expression and increasing VEGFA under hyperglycemia and hypoxia within a therapeutic window for myocardial infarction. Cardiovasc. Diabetol..

[13] Bakhashab S., Ahmed F., Schulten H.J., Ahmed F.W., Glanville M., Al-Qahtani M.H., Weaver J.U. (2018). Proangiogenic effect of metformin in endothelial cells is via upregulation of VEGFR1/2 and their signaling under hyperglycemia-hypoxia. Int. J. Mol. Sci..

[14] Ahmed F.W., Rider R., Glanville M., Narayanan K., Razvi S., Weaver J.U. (2016). Metformin improves circulating endothelial cells and endothelial progenitor cells in type 1 diabetes: MERIT study. Cardiovasc. Diabetol..

[15] Forouzandeh F., Salazar G., Patrushev N., Xiong S., Hilenski L., Fei B., Alexander R.W. (2014). Metformin beyond diabetes: Pleiotropic benefits of metformin in attenuation of atherosclerosis. J. Am. Heart Assoc..

[16] Mitchell P.S., Parkin R.K., Kroh E.M., Fritz B.R., Wyman S.K., Pogosova-Agadjanyan E.L., Peterson A., Noteboom J., O’Briant K.C., Allen A. (2008). Circulating microRNAs as stable blood-based markers for cancer detection. Proc. Natl. Acad. Sci. USA.

[17] Navickas R., Gal D., Laucevicius A., Taparauskaite A., Zdanyte M., Holvoet P. (2016). Identifying circulating microRNAs as biomarkers of cardiovascular disease: A systematic review. Cardiovasc. Res..

[18] Corsten M.F., Dennert R., Jochems S., Kuznetsova T., Devaux Y., Hofstra L., Wagner D.R., Staessen J.A., Heymans S., Schroen B. (2010). Circulating Microrna-208b and MicroRNA-499 reflect myocardial damage in cardiovascular disease. Circ. Cardiovasc. Genet..

[19] D’Alessandra Y., Devanna P., Limana F., Straino S., Di Carlo A., Brambilla P.G., Rubino M., Carena M.C., Spazzafumo L., De Simone M. (2010). Circulating microRNAs are new and sensitive biomarkers of myocardial infarction. Eur. Heart J..

[20] Kuwabara Y., Ono K., Horie T., Nishi H., Nagao K., Kinoshita M., Watanabe S., Baba O., Kojima Y., Shizuta S. (2011). Increased microRNA-1 and microRNA-133a levels in serum of patients with cardiovascular disease indicate myocardial damage. Circ. Cardiovasc. Genet..

[21] Jaguszewski M., Osipova J., Ghadri J.R., Napp L.C., Widera C., Franke J., Fijalkowski M., Nowak R., Fijalkowska M., Volkmann I. (2014). A signature of circulating microRNAs differentiates takotsubo cardiomyopathy from acute myocardial infarction. Eur. Heart J..

[22] Widera C., Gupta S.K., Lorenzen J.M., Bang C., Bauersachs J., Bethmann K., Kempf T., Wollert K.C., Thum T. (2011). Diagnostic and prognostic impact of six circulating microRNAs in acute coronary syndrome. J. Mol. Cell. Cardiol..

[23] Zhang R., Niu H., Ban T., Xu L., Li Y., Wang N., Sun L., Ai J., Yang B. (2013). Elevated plasma microRNA-1 predicts heart failure after acute myocardial infarction. Int. J. Cardiol..

[24] Eitel I., Adams V., Dieterich P., Fuernau G., de Waha S., Desch S., Schuler G., Thiele H. (2012). Relation of circulating MicroRNA-133a concentrations with myocardial damage and clinical prognosis in ST-elevation myocardial infarction. Am. Heart J..

[25] Osipova J., Fischer D.C., Dangwal S., Volkmann I., Widera C., Schwarz K., Lorenzen J.M., Schreiver C., Jacoby U., Heimhalt M. (2014). Diabetes-associated microRNAs in pediatric patients with type 1 diabetes mellitus: A cross-sectional cohort study. J. Clin. Endocrinol. Metab..

[26] Ortega F.J., Mercader J.M., Moreno-Navarrete J.M., Rovira O., Guerra E., Esteve E., Xifra G., Martinez C., Ricart W., Rieusset J. (2014). Profiling of circulating microRNAs reveals common microRNAs linked to type 2 diabetes that change with insulin sensitization. Diabetes Care.

[27] Vlachos I.S., Zagganas K., Paraskevopoulou M.D., Georgakilas G., Karagkouni D., Vergoulis T., Dalamagas T., Hatzigeorgiou A.G. (2015). DIANA-miRPath v3.0: Deciphering microRNA function with experimental support. Nucleic Acids Res..

[28] Salas-Perez F., Codner E., Valencia E., Pizarro C., Carrasco E., Perez-Bravo F. (2013). MicroRNAs miR-21a and miR-93 are down regulated in peripheral blood mononuclear cells (PBMCs) from patients with type 1 diabetes. Immunobiology.

[29] Olivieri F., Spazzafumo L., Bonafe M., Recchioni R., Prattichizzo F., Marcheselli F., Micolucci L., Mensa E., Giuliani A., Santini G. (2015). MiR-21-5p and miR-126a-3p levels in plasma and circulating angiogenic cells: Relationship with type 2 diabetes complications. Oncotarget.

[30] Zampetaki A., Kiechl S., Drozdov I., Willeit P., Mayr U., Prokopi M., Mayr A., Weger S., Oberhollenzer F., Bonora E. (2010). Plasma microRNA profiling reveals loss of endothelial miR-126 and other microRNAs in type 2 diabetes. Circ. Res..

[31] Cengiz M., Yavuzer S., Kilickiran Avci B., Yuruyen M., Yavuzer H., Dikici S.A., Karatas O.F., Ozen M., Uzun H., Ongen Z. (2015). Circulating miR-21 and eNOS in subclinical atherosclerosis in patients with hypertension. Clin. Exp. Hypertens..

[32] Darabi F., Aghaei M., Movahedian A., Pourmoghadas A., Sarrafzadegan N. (2016). The role of serum levels of microRNA-21 and matrix metalloproteinase-9 in patients with acute coronary syndrome. Mol. Cell. Biochem..

[33] Dey N., Das F., Mariappan M.M., Mandal C.C., Ghosh-Choudhury N., Kasinath B.S., Choudhury G.G. (2011). MicroRNA-21 orchestrates high glucose-induced signals to TOR complex 1, resulting in renal cell pathology in diabetes. J. Biol. Chem..

[34] Madhyastha R., Madhyastha H., Nakajima Y., Omura S., Maruyama M. (2012). MicroRNA signature in diabetic wound healing: Promotive role of miR-21 in fibroblast migration. Int. Wound J..

[35] Roy S., Khanna S., Hussain S.R., Biswas S., Azad A., Rink C., Gnyawali S., Shilo S., Nuovo G.J., Sen C.K. (2009). MicroRNA expression in response to murine myocardial infarction: miR-21 regulates fibroblast metalloprotease-2 via phosphatase and tensin homologue. Cardiovasc. Res..

[36] Raitoharju E., Lyytikainen L.P., Levula M., Oksala N., Mennander A., Tarkka M., Klopp N., Illig T., Kahonen M., Karhunen P.J. (2011). miR-21, miR-210, miR-34a, and miR-146a/b are up-regulated in human atherosclerotic plaques in the Tampere Vascular Study. Atherosclerosis.

[37] Sabatel C., Malvaux L., Bovy N., Deroanne C., Lambert V., Gonzalez M.L., Colige A., Rakic J.M., Noel A., Martial J.A. (2011). MicroRNA-21 exhibits antiangiogenic function by targeting RhoB expression in endothelial cells. PLoS ONE.

[38] Zhou J., Wang K.C., Wu W., Subramaniam S., Shyy J.Y., Chiu J.J., Li J.Y., Chien S. (2011). MicroRNA-21 targets peroxisome proliferators-activated receptor-alpha in an autoregulatory loop to modulate flow-induced endothelial inflammation. Proc. Natl. Acad. Sci. USA.

[39] Zuo K., Li M., Zhang X., Lu C., Wang S., Zhi K., He B. (2015). MiR-21 suppresses endothelial progenitor cell proliferation by activating the TGFbeta signaling pathway via downregulation of WWP1. Int. J. Clin. Exp. Pathol..

[40] Fleissner F., Jazbutyte V., Fiedler J., Gupta S.K., Yin X., Xu Q., Galuppo P., Kneitz S., Mayr M., Ertl G. (2010). Short communication: Asymmetric dimethylarginine impairs angiogenic progenitor cell function in patients with coronary artery disease through a microRNA-21-dependent mechanism. Circ. Res..

[41] Zhu S., Deng S., Ma Q., Zhang T., Jia C., Zhuo D., Yang F., Wei J., Wang L., Dykxhoorn D.M. (2013). MicroRNA-10A* and MicroRNA-21 modulate endothelial progenitor cell senescence via suppressing high-mobility group A2. Circ. Res..

[42] Ji R., Cheng Y., Yue J., Yang J., Liu X., Chen H., Dean D.B., Zhang C. (2007). MicroRNA expression signature and antisense-mediated depletion reveal an essential role of MicroRNA in vascular neointimal lesion formation. Circ. Res..

[43] Hu J.Z., Huang J.H., Zeng L., Wang G., Cao M., Lu H.B. (2013). Anti-apoptotic effect of microRNA-21 after contusion spinal cord injury in rats. J. Neurotrauma.

[44] Wang J., Gao Y., Duan L., Wei S., Liu J., Tian L., Quan J., Zhang Q., Liu J., Yang J. (2017). Metformin ameliorates skeletal muscle insulin resistance by inhibiting miR-21 expression in a high-fat dietary rat model. Oncotarget.

[45] Xiao H., Ma X., Feng W., Fu Y., Lu Z., Xu M., Shen Q., Zhu Y., Zhang Y. (2010). Metformin attenuates cardiac fibrosis by inhibiting the TGFbeta1-Smad3 signalling pathway. Cardiovasc. Res..

[46] Kim S.A., Choi H.C. (2012). Metformin inhibits inflammatory response via AMPK-PTEN pathway in vascular smooth muscle cells. Biochem. Biophys. Res. Commun..

[47] Chistiakov D.A., Sobenin I.A., Orekhov A.N., Bobryshev Y.V. (2015). Human miR-221/222 in physiological and atherosclerotic vascular remodeling. BioMed Res. Int..

[48] Dentelli P., Rosso A., Orso F., Olgasi C., Taverna D., Brizzi M.F. (2010). microRNA-222 controls neovascularization by regulating signal transducer and activator of transcription 5A expression. Arterioscler. Thromb. Vasc. Biol..

[49] Ihle J.N. (2001). The stat family in cytokine signaling. Curr. Opin. Cell Biol..

[50] Suarez Y., Fernandez-Hernando C., Pober J.S., Sessa W.C. (2007). Dicer dependent microRNAs regulate gene expression and functions in human endothelial cells. Circ. Res..

[51] Rippe C., Blimline M., Magerko K.A., Lawson B.R., LaRocca T.J., Donato A.J., Seals D.R. (2012). MicroRNA changes in human arterial endothelial cells with senescence: relation to apoptosis, eNOS and inflammation. Exp. Gerontol..

[52] Poliseno L., Tuccoli A., Mariani L., Evangelista M., Citti L., Woods K., Mercatanti A., Hammond S., Rainaldi G. (2006). MicroRNAs modulate the angiogenic properties of HUVECs. Blood.

[53] Tabasi S.A., Erson A.E. (2009). MIR222 (microRNA 222). Atlas Genet. Cytogenet. Oncol. Haematol..

[54] Liu X., Cheng Y., Yang J., Xu L., Zhang C. (2012). Cell-specific effects of miR-221/222 in vessels: Molecular mechanism and therapeutic application. J. Mol. Cell. Cardiol..

[55] McNamara C.A., Sarembock I.J., Bachhuber B.G., Stouffer G.A., Ragosta M., Barry W., Gimple L.W., Powers E.R., Owens G.K. (1996). Thrombin and vascular smooth muscle cell proliferation: Implications for atherosclerosis and restenosis. Semin. Thromb. Hemost..

[56] Akyurek L.M., Boehm M., Olive M., Zhou A.X., San H., Nabel E.G. (2010). Deficiency of cyclin-dependent kinase inhibitors p21Cip1 and p27Kip1 accelerates atherogenesis in apolipoprotein E-deficient mice. Biochem. Biophys. Res. Commun..

[57] Felli N., Fontana L., Pelosi E., Botta R., Bonci D., Facchiano F., Liuzzi F., Lulli V., Morsilli O., Santoro S. (2005). MicroRNAs 221 and 222 inhibit normal erythropoiesis and erythroleukemic cell growth via kit receptor down-modulation. Proc. Natl. Acad. Sci. USA.

[58] Minami Y., Satoh M., Maesawa C., Takahashi Y., Tabuchi T., Itoh T., Nakamura M. (2009). Effect of atorvastatin on microRNA 221/222 expression in endothelial progenitor cells obtained from patients with coronary artery disease. Eur. J. Clin. Investig..

[59] Coleman C.B., Lightell D.J., Moss S.C., Bates M., Parrino P.E., Woods T.C. (2013). Elevation of miR-221 and -222 in the internal mammary arteries of diabetic subjects and normalization with metformin. Mol. Cell. Endocrinol..

[60] Long G., Wang F., Duan Q., Yang S., Chen F., Gong W., Yang X., Wang Y., Chen C., Wang D.W. (2012). Circulating miR-30a, miR-195 and let-7b associated with acute myocardial infarction. PLoS ONE.

[61] Mortuza R., Feng B., Chakrabarti S. (2014). miR-195 regulates SIRT1-mediated changes in diabetic retinopathy. Diabetologia.

[62] Zheng D., Ma J., Yu Y., Li M., Ni R., Wang G., Chen R., Li J., Fan G.C., Lacefield J.C. (2015). Silencing of miR-195 reduces diabetic cardiomyopathy in C57BL/6 mice. Diabetologia.

[63] Okada M., Kim H.W., Matsu-ura K., Wang Y.G., Xu M., Ashraf M. (2016). Abrogation of age-induced microRNA-195 rejuvenates the senescent mesenchymal stem cells by reactivating telomerase. Stem Cells.

[64] Yang W.M., Jeong H.J., Park S.Y., Lee W. (2014). Saturated fatty acid-induced miR-195 impairs insulin signaling and glycogen metabolism in HepG2 cells. FEBS Lett..

[65] Arunachalam G., Samuel S.M., Marei I., Ding H., Triggle C.R. (2014). Metformin modulates hyperglycaemia-induced endothelial senescence and apoptosis through SIRT1. Br. J. Pharmacol..

[66] Orimo M., Minamino T., Miyauchi H., Tateno K., Okada S., Moriya J., Komuro I. (2009). Protective role of SIRT1 in diabetic vascular dysfunction. Arterioscler. Thromb. Vasc. Biol..

[67] Noren Hooten N., Martin-Montalvo A., Dluzen D.F., Zhang Y., Bernier M., Zonderman A.B., Becker K.G., Gorospe M., de Cabo R., Evans M.K. (2016). Metformin-mediated increase in DICER1 regulates microRNA expression and cellular senescence. Aging Cell.

[68] Bonetti P.O., Pumper G.M., Higano S.T., Holmes D.R., Kuvin J.T., Lerman A. (2004). Noninvasive identification of patients with early coronary atherosclerosis by assessment of digital reactive hyperemia. J. Am. Coll. Cardiol..

[69] Bustin S.A., Benes V., Garson J.A., Hellemans J., Huggett J., Kubista M., Mueller R., Nolan T., Pfaffl M.W., Shipley G.L. (2009). The MIQE guidelines: Minimum information for publication of quantitative real-time PCR experiments. Clin. Chem..

[70] Guescini M., Sisti D., Rocchi M.B., Stocchi L., Stocchi V. (2008). A new real-time PCR method to overcome significant quantitative inaccuracy due to slight amplification inhibition. BMC Bioinform..

