# Supplementary material for: Anti-Angiogenic miR-222, miR-195, and miR-21a Plasma Levels in T1DM Are Improved by Metformin Therapy, Thus Elucidating Its Cardioprotective Effect: The MERIT Study
Source: Int J Mol Sci. 2018 Oct 19;19(10):3242. doi: 10.3390/ijms19103242 (PMC6214022; doi:10.3390/ijms19103242)
Supplement: Supplementary file 1 [file ijms-19-03242-s001.pdf]

Supplementary Information

Article

Anti-angiogenic miR-222, miR-195, and miR-21a plasma levels in T1DM are Improved by Metformin Therapy, thus Elucidating its Cardioprotective Effect: The MERIT Study

Fahad W. Ahmed [1,2,3], Sherin Bakhashab [2,4,5], Inda T. Bastaman [1,2,6], Rachel E. Crossland [2], Michael Glanville [2] and Jolanta U. Weaver [1,2,7,*]

1 Department of Diabetes, Queen Elizabeth Hospital, Gateshead, Newcastle Upon Tyne NE9 6SH, UK; nfahadahmed@gmail.com (F.W.A.); itashab@gmail.com (I.T.B.)
2 Institute of Cellular Medicine, Newcastle University, Newcastle Upon Tyne NE2 4HH, UK; sbakhashab@kau.edu.sa (S.B.); rachel.crossland@newcastle.ac.uk (R.E.C.); michael.glanville@newcastle.ac.uk (M.C.)
3 Department of Diabetes and Endocrinology, Royal Sussex County Hospital, Brighton BN2 5BE, UK
4 Biochemistry Department, Faculty of Science, King Abdulaziz University, Jeddah P.O. Box 80218, Saudi Arabia
5 Center of Innovation in Personalized Medicine, King Abdulaziz University, Jeddah P.O. Box 80216, Saudi Arabia
6 Faculty of Medicine, Universitas Indonesia, Jakarta 10430, Indonesia
7 Cardiovascular Research Centre, Newcastle University, Newcastle Upon Tyne NE2 4HH, UK

* Correspondence: Jolanta.Weaver@newcastle.ac.uk; Tel.: +44-191-445-2181; Fax: +44-191-4456186

Trial registration: ISRCTN26092132

URL: http://controlled-trials.com/ISRCTN26092132 ; DOI: 10.1186/ISRCTN26092132

Supplementary Figures

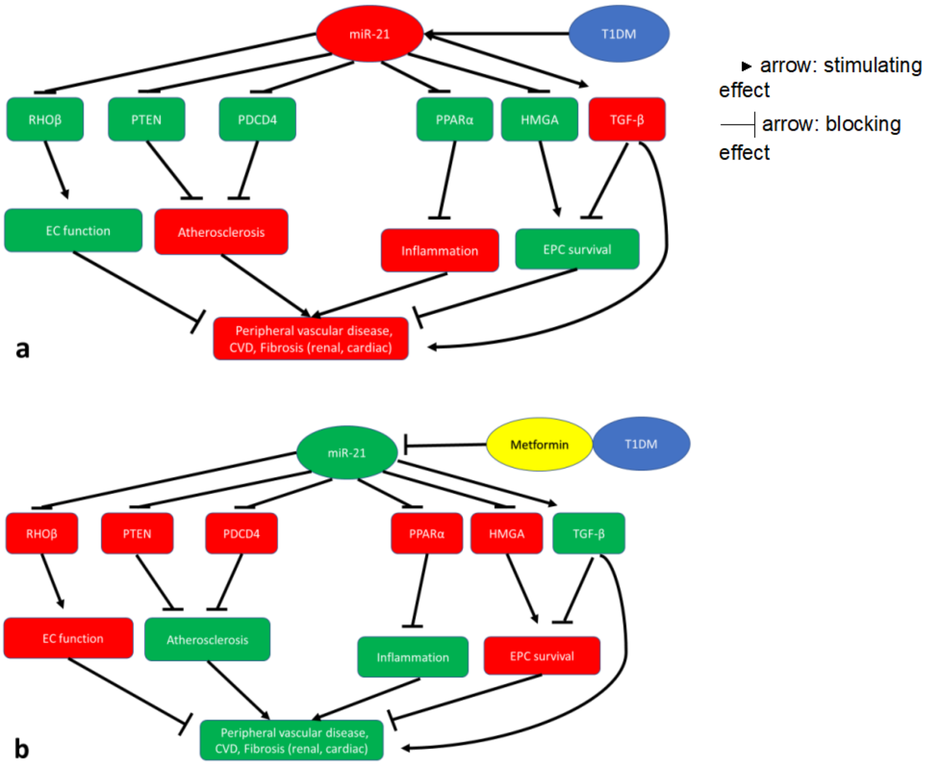

Supplementary Figure S1. Flowchart of the suggested effect of miR-21 in T1DM. a. Effect of miR-21 in the development of vascular pathology in T1DM patients. b. Effect of metformin on circulatory miR-21 levels. Green color represents downregulation, red color represents upregulation, blue color highlights T1DM and yellow metformin therapy.

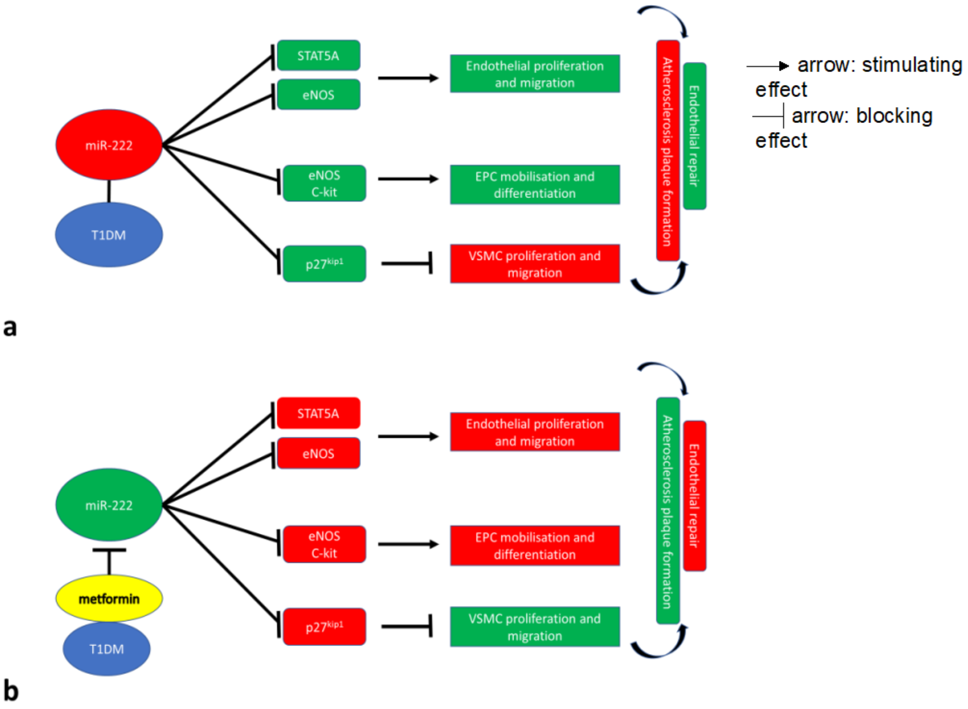

Supplementary Figure S2. Flowchart of the proposed effect of miR-222 in T1DM. a. Effect of miR-222 on the development of vascular pathology in T1DM patients. b. Effect of metformin on circulatory miR-222 levels. Green color represents down regulation, red color represents upregulation, blue color highlights T1DM and yellow metformin therapy.

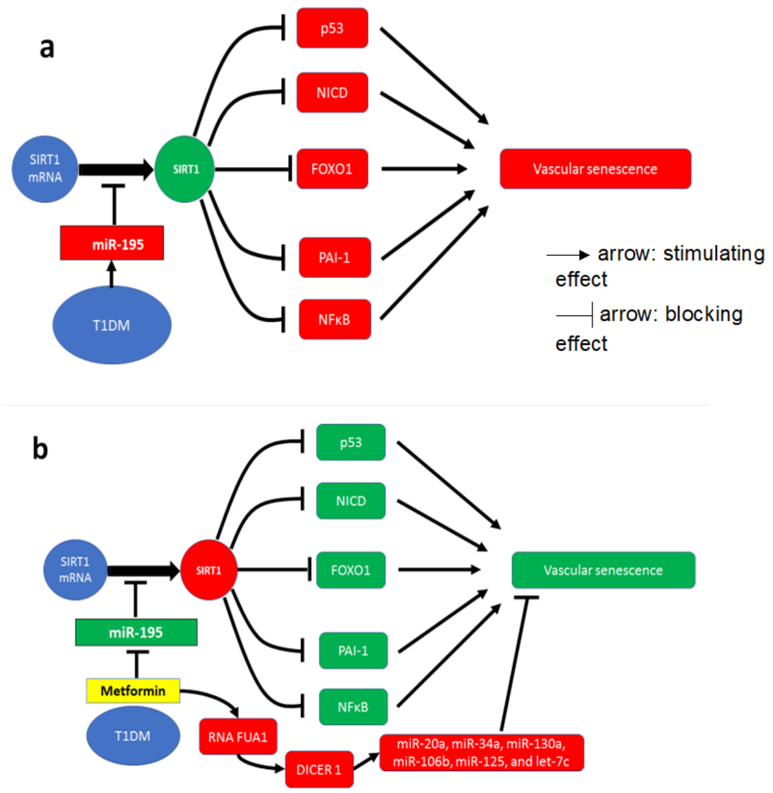

Supplementary Figure S3. Flowchart of the proposed effect of miR-195 in T1DM. a. The flowchart suggests the effect of miR-195 on the development of vascular pathology in T1DM patients. b. Effect of metformin on circulatory miR-195 levels. Green colour represents down regulation, red colour represents upregulation, blue colour highlights T1DM and yellow metformin therapy.
